# Comparative Effects of the Branched-Chain Amino Acids, Leucine, Isoleucine and Valine, on Gastric Emptying, Plasma Glucose, C-Peptide and Glucagon in Healthy Men

**DOI:** 10.3390/nu13051613

**Published:** 2021-05-11

**Authors:** Rachel A. Elovaris, Vida Bitarafan, Shahram Agah, Sina S. Ullrich, Kylie Lange, Michael Horowitz, Christine Feinle-Bisset

**Affiliations:** 1 Adelaide Medical School and Centre of Research Excellence in Translating Nutritional Science to Good Health, University of Adelaide, Adelaide 5005, Australia; rachel.elovaris@adelaide.edu.au (R.A.E.); vida.bitarafan@adelaide.edu.au (V.B.); shahram.agah@adelaide.edu.au (S.A.); kylie.lange@adelaide.edu.au (K.L.); michael.horowitz@adelaide.edu.au (M.H.); 2 Clinical Trials Unit, University Hospital Basel, 4031 Basel, Switzerland; sina.s.ullrich@gmail.com

**Keywords:** glucoregulatory hormones, glycaemia, insulin, human

## Abstract

(1) Background: Whey protein lowers postprandial blood glucose in health and type 2 diabetes, by stimulating insulin and incretin hormone secretion and slowing gastric emptying. The branched-chain amino acids, leucine, isoleucine and valine, abundant in whey, may mediate the glucoregulatory effects of whey. We investigated the comparative effects of intragastric administration of leucine, isoleucine and valine on the plasma glucose, C-peptide and glucagon responses to and gastric emptying of a mixed-nutrient drink in healthy men. (2) Methods: 15 healthy men (27 ± 3 y) received, on four separate occasions, in double-blind, randomised fashion, either 10 g of leucine, 10 g of isoleucine, 10 g of valine or control, intragastrically, 30 min before a mixed-nutrient drink. Plasma glucose, C-peptide and glucagon concentrations were measured before, and for 2 h following, the drink. Gastric emptying of the drink was quantified using ^13^C-acetate breath-testing. (3) Results: Amino acids alone did not affect plasma glucose or C-peptide, while isoleucine and valine, but not leucine, stimulated glucagon (*p* < 0.05), compared with control. After the drink, isoleucine and leucine reduced peak plasma glucose compared with both control and valine (all *p* < 0.05). Neither amino acid affected early (t = 0–30 min) postprandial C-peptide or glucagon. While there was no effect on overall gastric emptying, plasma glucose at t = 30 min correlated with early gastric emptying (*p* < 0.05). (4) Conclusion: In healthy individuals, leucine and isoleucine lower postprandial blood glucose, at least in part by slowing gastric emptying, while valine does not appear to have an effect, possibly due to glucagon stimulation.

## 1. Introduction

Ingestion of protein, particularly whey, lowers postprandial plasma glucose in healthy individuals and in people with type 2 diabetes (T2D) [1,2]. This beneficial effect of whey protein is associated with slowing of gastric emptying and the stimulation of insulin and the incretin hormones, glucagon-like peptide-1 (GLP-1) and glucose-dependent insulinotropic peptide (GIP) [2,3,4,5,6,7]. Gastric emptying is a key determinant of postprandial blood glucose concentrations, particularly the initial rise in blood glucose and slowing of gastric emptying reduces postprandial glucose excursions in both health and T2D [8,9,10]. These GI functions can be stimulated in a targeted fashion by providing nutrients in isolated form, i.e., not as part of a meal, as a so-called ‘preload’, approximately 30 min before a meal. For example, in diet-controlled T2D, acute ingestion of whey protein (55 g), 30 min prior to a carbohydrate meal, leads to a marked reduction in postprandial glucose, particularly within the first 30 min, associated with stimulation of insulin and slowing of gastric emptying [2].

The branched-chain amino acids, leucine, isoleucine and valine, which are abundant in whey protein, are potential mediators of the effects of protein on glucose homeostasis. Preclinical studies suggest that leucine and isoleucine regulate glycaemia by different mechanisms—leucine by stimulating insulin release via enhancing glutaminolysis, regulating gene transcription and protein synthesis [11] and isoleucine by increasing glucose uptake in skeletal muscle [12] and decreasing hepatic gluconeogenesis [13,14]. In humans, both leucine and isoleucine (in amounts of 8–10 g), coingested with 25 g glucose, attenuated the glycaemic response to the glucose load, although only leucine, but not isoleucine, stimulated insulin [15,16]. Moreover, intragastric administration of leucine and isoleucine (each at 10 g), 15 min before a mixed-nutrient drink (containing 56 g carbohydrates), reduced blood glucose modestly (1.1 mmol/L) [17]. Leucine stimulated insulin, while isoleucine slowed gastric emptying [17], indicating that the mechanisms underlying the glucose-lowering effects of the two amino acids differ. However, because the drink used in the latter study consisted primarily of fructose-based carbohydrates [17], it is not known whether the glucoregulatory responses to a drink containing glucose-based carbohydrates would differ.

There is limited, and inconsistent, evidence regarding the glycaemic effects of valine in both animals and humans [18,19,20]. In rats, acute oral administration of valine (in a dose of 1 g/kg body weight), 30 min prior to exercise, prevented the reduction in liver glycogen and blood glucose [18], consistent with an effect of valine to maintain blood glucose. In contrast, in rats, intrahypothalamic infusion of valine lowered circulating blood glucose comparably to leucine and isoleucine and, during a euglycaemic pancreatic clamp, reduced plasma glucose, apparently by decreasing liver glucose production [19]. Finally, in humans, 90-min intraduodenal infusion of valine, at loads of 0.15 and 0.45 kcal/min (administering a total of 3.3 g and 9.9 g, respectively), had no effect on fasting blood glucose [20], in contrast to intraduodenal leucine [21]. Accordingly, the effects of valine on postprandial blood glucose remain uncertain.

The aim of the current study was to determine the comparative effects of leucine, isoleucine and valine on the blood glucose, C-peptide (as a measure of insulin secretion) and glucagon responses to and gastric emptying of a mixed-nutrient drink, containing maltodextrin and sucrose, in healthy men. Amino acids were administered intragastrically to avoid potential confounding effects due to their unpleasant taste.

## 2. Materials and Methods

### 2.1. Study Participants

Fifteen healthy males, aged 27 ± 3 years (range: 20–51 years) and of normal body weight (body mass index 23 ± 0.5 kg/m^2^ (range: 20–25 kg/m^2^)), participated in the study. The number of participants was determined by power calculations derived from our previous study [17]. We calculated that *n* = 15 participants would allow detection of a 1.2 mmol/L reduction in plasma glucose, assuming a within-subjects standard deviation of 1.1 mmol/L, at α = 0.05, with a power of 80%. Participants were recruited through advertisements on online sites (University of Adelaide and Gumtree) and at the University of Adelaide, University of South Australia, Flinders University and Royal Adelaide Hospital, and from an existing pool of volunteers. All participants were unrestrained eaters (score ≤12 on the eating restraint component of the three-factor eating questionnaire [22]) and had been weight-stable (<5% fluctuation) in the 3 months preceding the study. Individuals who smoked, consumed >20 g alcohol/day, had low ferritin (<30 ug/L) or iron (<8 umol/L) concentrations (a requirement of our Ethics Committee), were lactose-intolerant, vegetarians or high-performance athletes, had significant gastrointestinal (GI) symptoms, disease or surgery or used medications known to affect GI functions and/or appetite were excluded. Once included, participants were allocated a treatment order generated using an online tool (www.randomization.com (accessed on 11 May 2021)) with balanced permutations by a researcher who was not involved in the data analysis. The study protocol was approved by the Human Research Ethics Committee of the Central Adelaide Local Health Network, and the study performed in accordance with the Declaration of Helsinki and the NHMRC Statement on Ethical Conduct in Human Research. All participants provided written, informed consent prior to their enrolment. The study was registered as a clinical trial with the Australian New Zealand Clinical Trials Registry (www.anzctr.org.au (accessed on 11 May 2021), number: ACTRN12619000718145).

### 2.2. Study Design

**Figure 1:**
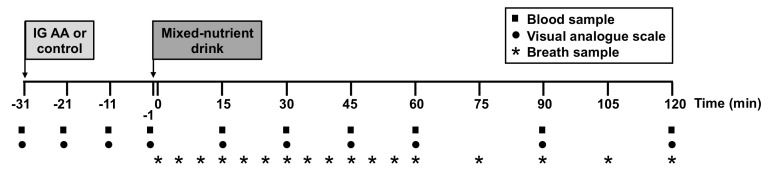
Schematic representation of the study design. At t = −31 min, a baseline blood sample (for measurement of plasma glucose and glucoregulatory hormones) was collected and a visual analogue scale questionnaire (to assess GI symptoms) completed, then study treatments (either an amino acid (AA; leucine, isoleucine or valine) or control) were administered intragastrically within 1 min. After 30 min, at t = −1 min, each participant consumed, within 1 min, 350 mL of a mixed-nutrient drink, containing 100 mg of ^13^C-acetate for measurement of gastric emptying by ^13^CO_2_ breath test. Blood samples and visual analogue ratings were collected at the indicated time points throughout the study, and breath samples after the mixed-nutrient drink.

The study evaluated the effects of intragastric administration of 10 g of leucine, 10 g of isoleucine or 10 g of valine, or control, on the plasma glucose, C-peptide and glucagon responses to and the gastric emptying of a mixed-nutrient carbohydrate-containing drink consumed 30 min later (Figure 1). The dose of the amino acids and the 30 min between treatments and the drink were based in our previous studies, in which nutrients stimulated glucoregulatory hormones within this time [17,20,21].

### 2.3. Study Treatments

Due to the low water solubility (particularly of leucine), amino acids were administered as suspensions at room temperature. Of crystalline leucine (Bulk Nutrients, Tasmania, Australia), isoleucine or valine (both from PureBulk Inc., Roseburg, OR, USA) 10 g of and 29.2 mg of CaCl_2_ × 2H_2_O were incorporated in 10 mL of a suspending agent (ORA-Plus^®^, Perrigo^®^, Minneapolis, MN, USA), and isotonic saline was used to adjust to a final volume of 100 mL. The control treatment consisted of 10 mL suspending agent, 29.2 mg of CaCl_2_ × 2H_2_O and 90 mL of isotonic saline. Suspensions were prepared on the morning of each study visit by a research officer who was not involved in the performance of the study or analysis of the data. Syringes were covered so that both the study participant and primary investigator (RAE) were blinded to the treatments.

### 2.4. Study Protocol

Each participant was studied on 4 occasions, separated by at least 3, and up to 7, days. Participants were instructed to abstain from vigorous exercise and alcohol intake for 24 h before each study visit and provided with a standardised evening meal (beef lasagne; energy content: 602 kcal; McCain Food, Wendouree, Victoria, Australia) to be consumed by 7:00 p.m. on the night before each study. Participants were instructed not to consume any other solid foods or liquids (with the exception of water, which was allowed until 6:30 a.m.) until they arrived at the Clinical Research Facility at the University of Adelaide at 8:30 a.m. the following morning. Upon arrival they were seated in an upright position, and an intravenous cannula was placed into a forearm vein for regular blood sampling. At baseline (t = −31 min), a blood sample to assess plasma glucose and hormone concentrations, and a breath sample to measure gastric emptying, were collected, and the participant completed a visual analogue scale (VAS) questionnaire to assess GI symptoms. Participants were then intubated with a nasogastric, soft-silicone feeding tube (outer diameter: 4 mm; Dentsleeve International, Mississauga, ON, Canada), which was inserted through an anaesthetised nostril into the stomach. The correct positioning of the tube was checked by auscultating the stomach with the use of a stethoscope while pushing a 5-mL air bolus through the tube. Immediately thereafter, participants received, into the stomach, one of the study treatments, within 1 min. The tube was then removed, and blood samples and VAS questionnaires were collected every 10 min for the next 30 min (t = −31 to −1 min). At t = −1 min, each participant consumed, within 1 min, 350 mL of a mixed-nutrient drink (Resource Plus^®^; Nestle, Tongala, Victoria, Australia (325 mL); 500 kcal; 74 g carbohydrates, including maltodextrin and sucrose, 18 g protein, 15 g fat; plus 25 mL water to make up the final volume) containing 100 mg ^13^C-acetate for measurement of gastric emptying by breath-test [23]. After consumption of the drink, VAS questionnaires and blood samples were collected at 15-min intervals for the next hour (t = 15–60 min). For the second hour (t = 60–120 min), VAS questionnaires and blood samples were collected every 30 min. Breath samples were collected every 5 min for the first hour and every 15 min subsequently. At t = 120 min, the cannula was removed, and participants were presented with a light lunch, after which they were free to leave the laboratory.

### 2.5. Measurements

#### 2.5.1. Plasma Glucose and Hormone Analyses

Blood samples were collected into ice-chilled ethylenediaminetetraacetic acid-coated tubes. Plasma was obtained by centrifugation at 3200× *g* (1832 × *g*-force) for 15 min at 4 °C within 15 min of collection and then stored at −80 °C for subsequent analysis.

Plasma glucose (mmol/L) was measured using the glucose oxidase technique (2300 STAT Plus; YSI, Yellow Springs, OH, USA).

Plasma C-peptide concentrations (expressed as pmol/L) were measured by ELISA (10-1113; Mercodia, Uppsala, Sweden). The minimal detectable limit was 15 pmol/L, and intra-assay and inter-assay CVs were 2.4% and 4.9%, respectively.

Plasma glucagon (pg/mL) was measured by radioimmunoassay (GL-32K; Millipore, Billerica, MA, USA). The minimum detectable limit was 15 pg/mL, and intra-assay and inter-assay CVs were 6.9% and 4.2%, respectively.

#### 2.5.2. Gastric Emptying

Gastric emptying was measured by breath-test using ^13^C-acetate [23]. ^13^CO_2_ concentrations in exhaled air of end-expiratory breath samples were measured using an isotope ratio mass spectrometer (FANci2 breath test analyser, Fischer Analysen Instrumente, Leipzig, Germany) with an online gas chromatographic purification system. Breath sample data were expressed as the percentage of recovery of ^13^CO_2_ in the breath per hour.

#### 2.5.3. GI Symptoms

Nausea and bloating were assessed using validated 100-mm VAS questionnaires [24]. VAS scales consisted of 100-mm horizontal lines, where 0 mm represented “not felt at all”, and 100 mm “felt the strongest possible”. Participants were asked to place a vertical mark on each horizontal line to rate the strength of each sensation felt at that point in time.

### 2.6. Data and Statistical Analysis

Statistical analysis was performed with SPSS software (version 26.0; IBM, Chicago, IL, USA). Plasma glucose, C-peptide and glucagon concentrations and gastric emptying were expressed as absolute values, and VAS data as changes from baseline (i.e., t = −31 min), to account for variations in baseline values.

Effects of amino acids alone (i.e., prior to ingestion of the mixed-nutrient drink) on plasma glucose, C-peptide and glucagon concentrations and VAS symptom ratings were evaluated by repeated-measures two-way analysis of variance (ANOVAs) with treatment (leucine, isoleucine, valine and control) and time (t = −31–−1 min) as factors. Plasma glucose and hormone data were also analysed as total area under the curve (AUC) (expressed as AUC_−31–−1 min_), using repeated-measure one-way ANOVA with treatment as a factor. To evaluate responses to the mixed-nutrient drink, plasma glucose, C-peptide and glucagon concentrations and symptom ratings were analysed using repeated-measures two-way ANOVAs with treatment and time (t = 15–120 min), as factors. The data were also summarised as total AUC (expressed as AUC_15–120 min_) and analysed using repeated-measures one-way ANOVA with treatment as a factor. Peak plasma glucose was analysed using repeated-measures one-way ANOVA with treatment as a factor. Gastric emptying was analysed as AUC_0–120 min_. Post-hoc comparisons, adjusted for multiple comparisons by Bonferroni’s correction, were performed where ANOVAs revealed significant effects. Sphericity of the repeated effects was evaluated by Mauchly’s test and, when violated, the adjusted Greenhouse–Geisser *p*-value was reported. Following examination of the data, post-hoc analyses were also conducted over the first 30 min post-meal for C-peptide, glucagon (both expressed as AUC_−1–30 min_) and gastric emptying (expressed as AUC_0–30 min_), representing ‘early changes’. Correlations between plasma glucose at t = 30 min with early changes in plasma C-peptide and glucagon and gastric emptying were evaluated, using data across all study days, with the use of linear within-subject correlations [25]. All data are reported as means ± SEMs. All tests were two-tailed, and differences were considered statistically significant at *p* ≤ 0.05.

## 3. Results

All participants tolerated the study treatments well and completed all study visits without reporting any adverse effects.

### 3.1. Plasma Glucose Concentrations

**Figure 2:**
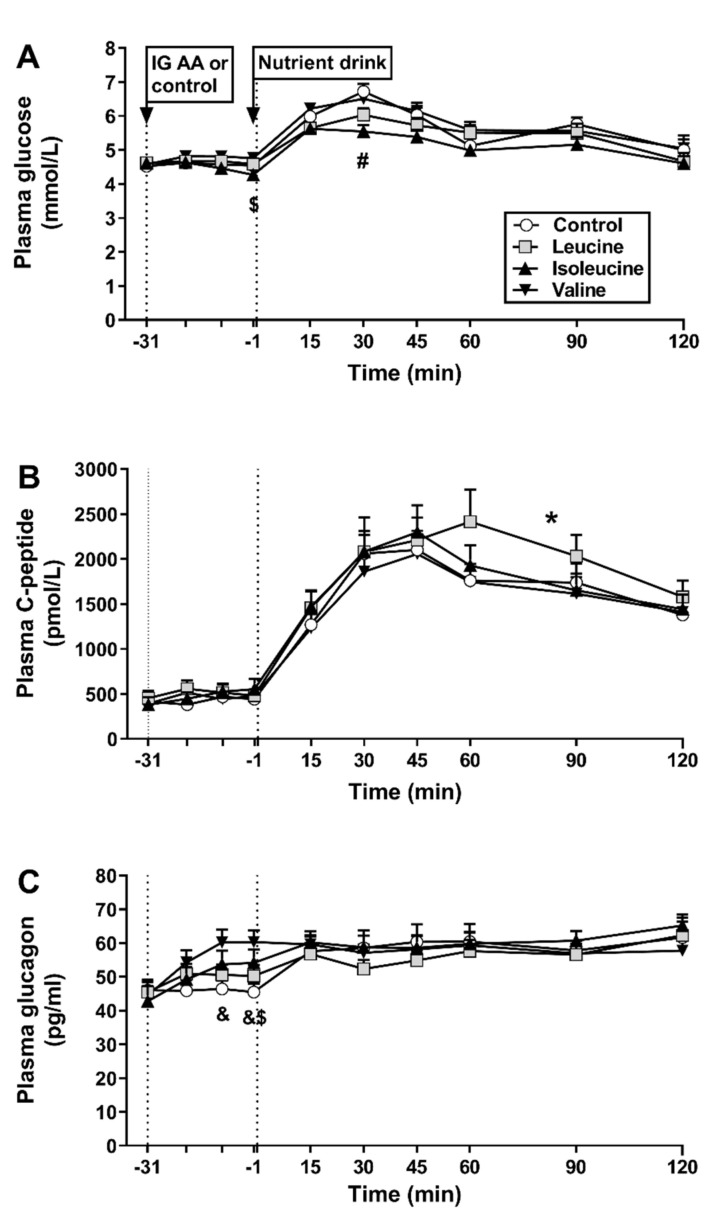
Plasma glucose (**A**), C-peptide (**B**) and glucagon (**C**) concentrations at baseline (t = −31 min), in response to intragastric (IG) administration of either 10 g of leucine, 10 g of isoleucine, 10 g of valine or control (t = −20, −10 and −1 min), and after a mixed-nutrient drink (t = 15–120 min). Data were analysed using repeated-measures two-way ANOVA with treatment and time as factors. Post-hoc comparisons, adjusted for multiple comparisons by Bonferroni’s correction, were conducted where ANOVAs revealed significant effects. (**A**) $ isoleucine significantly different from valine at t = −1 min, *p* < 0.05; # leucine and isoleucine significantly different from both control and valine, both *p* < 0.05. (**B**) * leucine, but not isoleucine or valine, significantly different from control, *p* < 0.05. (**C**) & valine significantly different at t = −10 and −1 min, and $ isoleucine at t = −1 min, from control, all *p* < 0.05. Data are means ± SEMs; *n* = 15; AA, amino acid.

There were no differences in baseline (fasting) concentrations of plasma glucose between study days (Figure 2A).

Response to amino acids alone: There was a treatment × time interaction for plasma glucose (*p* < 0.05); isoleucine reduced plasma glucose at t = −1 min, compared with valine (*p* < 0.05), while there was no difference between amino acids and control (Figure 2A). There was a trend for an effect of treatment on plasma glucose AUC_−31–−1 min_ (*p* = 0.098; Table 1).

Response to the mixed-nutrient drink: Plasma glucose increased on all study days, with a maximum concentration of 7 mmol/L on the control day, and <7 mmol/L on amino acid days. There was a treatment × time interaction for plasma glucose (*p* < 0.05); isoleucine reduced (*p* < 0.05), and leucine tended to reduce (*p* = 0.055), plasma glucose at t = 30 min, compared with control, while there was no difference between valine and control. Moreover, isoleucine at t = 15–60 min and leucine at t =15 min reduced blood glucose compared with valine (all *p* < 0.05) (Figure 2A). There was also an effect of treatment on peak plasma glucose (*p* < 0.05); leucine and isoleucine reduced peak glucose, compared with both control and valine (*p* < 0.05, control: 7.0 ± 0.2 mmol/L, leucine: 6.3 ± 0.2 mmol/L, isoleucine: 6.0 ± 0.1 mmol/L, valine: 6.8 ± 0.2 mmol/L). Finally, there was an effect of treatment on plasma glucose AUC_15–120 min_ (*p* = 0.001); isoleucine reduced AUC_15–120 min_ compared with control and valine (*p* < 0.05) (Table 1).

### 3.2. Plasma C-Peptide Concentrations

There were no differences in baseline C-peptide concentrations between study days (Figure 2B).

Response to amino acids alone: There was no effect of treatment or time on plasma C-peptide (Figure 2B) or on AUC_−31–−1 min_ (Table 1).

Response to the mixed-nutrient drink: There were effects of treatment and time, but no interaction, on overall plasma C-peptide (*p* < 0.05). Plasma C-peptide increased markedly on all study days. Leucine, but not isoleucine or valine, increased C-peptide, compared with control (*p* < 0.05). There were no effects of treatment or time, or an interaction, on plasma C-peptide over the first 30 min post-drink (Figure 2B). There was an effect of treatment on plasma C-peptide AUC_15–120 min_ (*p* = 0.001), but not on AUC_−1–30 min_; AUC_15–120 min_ was greater after leucine compared with control, isoleucine and valine (Table 1).

### 3.3. Plasma Glucagon Concentrations

There were no differences in baseline glucagon concentrations between study days (Figure 2C).

Response to amino acids alone: There was a treatment × time interaction for plasma glucagon (*p* < 0.05); valine increased glucagon at t = −10 and −1 min, and isoleucine at t = −1 min (all *p* < 0.05), compared with control, while there was no difference between leucine and control (Figure 2C). There was also an effect of treatment on plasma glucagon AUC_−31–−1 min_ (*p* = 0.006); there was a trend for plasma glucagon to be greater after valine compared with control (*p* = 0.061; Table 1).

Response to the mixed-nutrient drink: There were no effects of treatment or time, or an interaction, on overall plasma glucagon or during the first 30 min post-drink, or on AUC_−1–30 min_ or AUC_15–120 min_.

### 3.4. Gastric Emptying

**Figure 3:**
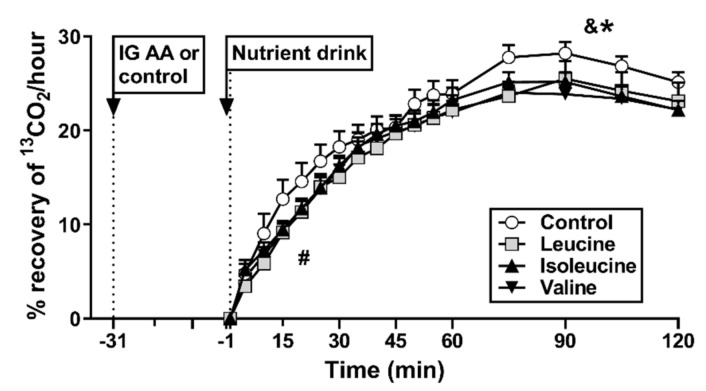
Recovery of ^13^CO_2_ in breath samples as a measure of gastric emptying of a mixed-nutrient drink, containing 100 mg ^13^C-acetate, consumed at t = −1 min, 30 min after intragastric (IG) administration of either 10 g of leucine, 10 g of isoleucine, 10 g of valine or control. Gastric emptying was expressed as AUC_0–120 min_ and AUC_0–30 min_. * Leucine AUC_0–120 min_ significantly different from control, *p* < 0.05, & trend for valine to differ from control, *p* = 0.051. # Trend for an effect of treatment on early gastric emptying (i.e., t = 0–30 min post-drink), *p* = 0.096. Data are means ± SEMs; *n* = 15; AA, amino acid.

There was an effect of treatment on overall gastric emptying (i.e., AUC_0–120 min_) of the drink (*p* < 0.05). Leucine, but not isoleucine, slowed (*p* < 0.05), and valine tended to slow (*p* = 0.051), gastric emptying, compared with control (Figure 3). There was a trend for an effect of treatment on early gastric emptying (i.e., t = 0–30 min post-drink; AUC_0–30 min_) (*p* = 0.096).

### 3.5. Gastrointestinal Symptoms

There were no differences in baseline ratings, or any effects of treatment, on nausea or bloating (data not shown).

### 3.6. Relationships between Plasma Glucose with C-Peptide, Glucagon or Gastric Emptying

There were no relationships between plasma glucose at t = 30 min with early C-peptide AUC_−1–30 min_ (r = 0.1, *p* = 0.3) or glucagon AUC_−1–30 min_ (r = 0.08, *p* = 0.5), but a positive correlation with early gastric emptying AUC_0–30 min_ (r = 0.35, *p* < 0.01).

## 4. Discussion

Our study shows that both leucine and isoleucine when administered intragastrically, in a dose of 10 g, in healthy individuals, modestly reduced peak glucose in response to a mixed-nutrient drink, while valine had no effect. None of the amino acids affected early plasma C-peptide, a marker of insulin secretion. Valine and, to a lesser extent, isoleucine, but not leucine, stimulated glucagon before the drink. While all three amino acids appeared to have a small, albeit non-significant, effect to slow early gastric emptying, this was only associated with postprandial glucose lowering after leucine and isoleucine. The absence of an effect of valine to lower postprandial blood glucose may reflect its effect to stimulate glucagon.

That both leucine and isoleucine, at the dose of 10 g, modestly lowered blood glucose after a carbohydrate-containing drink, is consistent with previous findings by ourselves [17,21] and others [15,16]. The effects of the branched-chain amino acid, valine, on the postprandial glucose response to a mixed-nutrient drink have, to our knowledge, not been investigated previously in humans.

While the magnitude of the effects of leucine and isoleucine to lower the blood glucose response to a drink containing primarily glucose-based carbohydrates is of the same order as their glucose-lowering effect in response to a predominantly fructose-based drink in our previous study [17], it appears that the underlying mechanisms may differ between the two test drinks. In our previous study, leucine stimulated C-peptide within the first 60 postprandial minutes and did not slow gastric emptying, while isoleucine slowed gastric emptying, albeit only after the first 30 postprandial minutes, but did not stimulate C-peptide. In the current study, while leucine stimulated C-peptide, this effect was apparently due to an increase in the second postprandial hour, indicating that the effect of leucine to lower blood glucose, which was maximal at 30 min post-drink, was unrelated to C-peptide stimulation. In contrast to our previous study [17], leucine slowed gastric emptying of the drink, including a trend for slowing of early gastric emptying (i.e., within the first 30 min post-drink), which probably contributed to blood glucose-lowering, as supported by the observed correlation between blood glucose concentrations at 30 min and early gastric emptying. It is not clear why leucine would slow gastric emptying of a drink containing predominantly glucose-, but not fructose-, based carbohydrates, but the observed differences may potentially reflect differential interactions with other macronutrients, which warrants further investigation. The effect of leucine to modestly slow early gastric emptying, even though it was not statistically significant, may account for the absence of a stimulatory effect on early C-peptide secretion.

In line with previous observations by ourselves [17] and others [16], isoleucine did not stimulate C-peptide. In contrast, there was a trend for an effect to slow early gastric emptying. We demonstrated that even small changes in the rate of glucose delivery to the small intestine can have a major impact on blood glucose concentrations [26]. Thus, the observed effect of isoleucine (and leucine) to lower postprandial blood glucose concentrations may be related to their effect to slow early gastric emptying.

To our knowledge, this is the first study to evaluate the postprandial glycaemic effects of valine in humans. Unlike leucine and isoleucine, valine does not appear to have glucose-lowering effects. Valine did not stimulate C-peptide, may have a modest (although statistically non-significant) effect to slow early gastric emptying and potently stimulated glucagon prior to drink ingestion. While it is unknown whether valine may selectively stimulate pancreatic alpha-cells [27], valine-induced glucagon stimulation would counteract any potential glucose-lowering due to slowing of gastric emptying.

Given that both leucine and isoleucine have been shown to have modest effects to lower postprandial blood glucose in both the current and previous [15,16,17] studies, it is important to consider the potential clinical implications of these findings for the management, treatment or prevention of T2D. It is tempting to hypothesise that if these amino acids lower blood glucose in healthy people with good blood glucose control, it is likely that these effects may even be more pronounced in patients with T2D that have elevated blood glucose concentrations [17]. However, we have reported recently [28], using an identical study design, that leucine and isoleucine, in the same doses, did not lower the blood glucose response to a mixed-nutrient drink in people with T2D. Thus, the glucose-lowering effect of these amino acids appears not to be maintained, arguing against the use of leucine or isoleucine as a nutrient-based strategy in the management of T2D.

A number of limitations for this study should be recognised. We studied only healthy participants, thus, our results cannot be extended to people with impaired glucose tolerance or T2D. Whether the effect of leucine and isoleucine may be greater in individuals with impaired glucose tolerance or T2D, due to elevated blood glucose levels, warrants further investigation. While the 10 g of dose of each amino acid used is relatively high, it is representative of what may be consumed in a meal, given that there are relatively large amounts of leucine and isoleucine, and somewhat less valine, in foods, including beef, chicken and tuna (e.g., approximately 10 g of leucine is contained in a 350-g steak). We were unable to evaluate the effect of oral ingestion of the amino acids, due to their unpleasant taste. Finally, we did not assess plasma amino acid concentrations, however, it is known from studies by ourselves [21] and others [15,16] that plasma concentrations of these amino acids rise within 15–30 min of administration of preloads of whey [29,30] or individual amino acids [15,16,21].

In conclusion, among the branched-chain amino acids, leucine and isoleucine, but not valine, modestly diminish the blood glucose response after a mixed-nutrient drink containing glucose-based carbohydrates in healthy people, most likely due to modest slowing of early gastric emptying.

## Figures and Tables

**Table 1:** Effects of intragastric administration of the branched-chain amino acids, leucine, isoleucine or valine, or control on plasma glucose, C-peptide and glucagon concentrations and gastric emptying ^1^.

Unit	Control	Leucine	Isoleucine	Valine	ANOVA *p*-Value
Plasma glucose					
AUC_−31–−1 min_, mmol/L × min	137 ± 3	135 ± 1	139 ± 2	143 ± 2	0.098
AUC_15–120 min_, mmol/L × min	678 ± 14	654 ± 20	616 ± 17*	687 ± 24 ^#^	0.001
Plasma C-peptide					
AUC_−31–−1 min_, pmol/L × min	12,806 ± 1783	15,406 ± 2339	14,397 ± 2281	13,926 ± 1818	0.302
AUC_−1–30 min_, pmol/L × min	37,840 ± 4266	41,092 ± 4903	41,803 ± 5821	35,996 ± 4471	0.333
AUC_15–120 min_, pmol/L × min	184,431 ± 17,969	214,318 ± 23,924 *^#$^	191,239 ± 22,908	176,874 ± 16,504	0.001
Plasma glucagon					
AUC_−31–−1 min_, pg/mL × min	0 ± 87	98 ± 73	231 ± 53	333 ± 104 ^@^	0.006
AUC_−1–30 min_, pg/mL × min	180 ± 75	116 ± 39	250 ± 46	208 ± 50	0.332
AUC_15–120 min_, pg/mL × min	1400 ± 587	1053 ± 301	1864 ± 351	1413 ± 355	0.537
Gastric emptying					
AUC_0–30 min_, % recovery of ^13^CO_2_ × min	327 ± 49	249 ± 26	290 ± 23	261 ± 24	0.096
AUC_0–120 min_, % recovery of ^13^CO_2_ × min	2555 ± 116	2169 ± 118 *	2335 ± 100	2229 ± 90 ^%^	0.004

^1^ Data are means ± SEMs. AUC, area under the curve. All parameters are expressed as absolute data and were analysed using repeated-measures one-way ANOVA with treatment as a factor. Post-hoc comparisons, adjusted for multiple comparisons by Bonferroni’s correction, were conducted where ANOVAs revealed significant effects. * Significantly different from control (*p* < 0.05); ^#^ significantly different from isoleucine (*p* < 0.05); ^$^ significantly different from valine (*p* < 0.05); ^@^ trend for a difference from control (*p* = 0.061); ^%^ trend for a difference from control (*p* = 0.051).

## Data Availability

The data described in the manuscript will be made available (in de-identified form) upon request.
